# Mechanistic role of alpha oscillations in a computational model of working memory

**DOI:** 10.1371/journal.pone.0296217

**Published:** 2024-02-08

**Authors:** Gustavo Soroka, Marco Idiart, Aline Villavicencio

**Affiliations:** 1 Neuroscience Graduate Program, Federal University of Rio Grande do Sul (UFRGS), Porto Alegre, Brazil; 2 Department of Physics, Institute of Physics, Federal University of Rio Grande do Sul (UFRGS), Porto Alegre, Brazil; 3 Department of Computer Science, University of Sheffield, Sheffield, United Kingdom; Sorbonne Universite UFR de Biologie, FRANCE

## Abstract

Brain oscillations are believed to be involved in the different operations necessary to manipulate information during working memory tasks. We propose a mechanistic role for the observed inhibition effect of the alpha rhythm based on its interference with the theta rhythm. Using the Lisman-Idiart model for multi-item working memory, we show that the interaction between these two oscillations is capable of creating a long lasting destructive interference that prevents the cyclic reactivation of neuronal ensembles and, as a consequence, memory maintenance. Additionally, to ensure robustness we propose a modular version of the model and implement oscillations as traveling waves. Using this model, we show that the interactions between theta and gamma determine the allocation of multiple memories in distinct modules, while the interference between theta and alpha disrupts the maintenance of the information already stored in them. The effect of alpha in erasing or blocking storage is robust and seems fairly independent of frequency, as long as it stays within the alpha range. This model helps us to understand why the alpha and theta oscillations, which have close frequency bands, could have opposite roles in working memory.

## Introduction

Working memory is a putative memory system that incorporates many short-term information storage subsystems [1, 2], and serves as an interface between perception, long term memory and action [3]. In doing so, it contributes to higher cognitive functions such as reasoning, planning, decision-making and language comprehension [4].

Comprehensive models for a working memory system include specific components, or subsystems, to deal with different kinds of information and processes [2, 5, 6]. In particular, for the short-term storage devices that compose the working memory system, most of the proposed models agree that some sort of persistent activity of the neurons involved in storing the information is necessary, but disagree on the underlying physiological mechanism. These models can be divided in roughly two categories: those that favor network mechanisms (e.g. instantaneous attractors due to short-term synaptic changes and continuous attractors or bump models) and models that favor single cell phenomena.

In the last category is the model proposed by Lisman and Idiart [7]. According to this model, incoming information of a sequence of items to be memorized causes the ordered activation of item specific neural ensembles. The firing of these cells changes their intrinsic membrane properties producing a transitory excitability which peaks on a time scale of approximately 120ms after firing. In the absence of external inputs, these labeled cells can be reactivated by a nonspecific (non-informative) oscillatory input, provided that their period corresponds to the characteristic time of transient excitability. Therefore, they should be in the theta range. Another specific requirement of the model is feedback inhibition that prevents the synchronization of the different ensembles, so that the individual items are held active in time multiplexed fashion. A prediction of this model is a phase-amplitude coupling between the slow oscillations in the theta range representing the maintenance signal and the fast oscillations in the gamma range representing the firing of the stored memories. Subsequent studies have explored the theta-gamma mechanism in different models [8, 9] (see [10] for a review).

Another oscillation that has been associated with an active role in cognitive processes, including working memory, is the alpha oscillation, which was the first rhythm observed in humans almost a century ago [11]. Increases in alpha power were related to inhibited activity of areas during working memory maintenance tasks (see [12] for a review). During working memory scanning tasks, where subjects need to hold for a brief period of time several items in memory which are later tested, an increase in alpha power was observed during the retention period while a decrease was observed during the retrieval of information [13–15]. An increase in alpha power related to the number of stored items was also observed [13–15].

A few theories have been used to describe possible roles for the alpha oscillation. The *inhibition-timing hypothesis* proposes that the peaks of alpha constrains time-windows of opportunities for the firing of neurons [16], with the increase of alpha power leading to a decrease of the windows size. The *gating by inhibition hypothesis* supposes that alpha can select the most relevant information to be processed through the blocking of irrelevant information routes [17]. The *oscillatory selection hypothesis* suggests that the information selection could be accomplished by an entrainment between sensory stimulus and brain oscillations [18]. In general, the main functional role addressed for alpha oscillations relates to the inhibition of irrelevant information.

Dippopa et. al. [19, 20] proposed a computational model where brain oscillations act as functional operators of a working memory network, in other words, applying external oscillatory currents to the network allows it to store, maintain, prevent upload and erase information. In the model, oscillations in the beta-gamma range allow the upload of information, oscillations in the alpha range are responsible for erasing the content and preventing new content from being uploaded to the network, while oscillations in the theta range block upload but maintain the current content. However, in their current version of the model the issue of multiple item memory as well as the role of the oscillations phases are not fully explored.

Inspired by Dippopa et. al. [19], in this article we theoretically explore the possibility that oscillations in different frequency bands can play different roles in storage in a model with the characteristics of the Lisman-Idiart short-term memory model [7]. More specifically, we want to explore whether increasing alpha power at the external oscillatory input can disrupt the multiple-item maintenance in the model therefore acting as a gating signal to erase and block memory storage.

The Lisman-Idiart model hinges on three essential elements: neural short-term excitability, concurrent external oscillation, and feedback inhibition. This paper, similar to the work of Dipoppa and Gutkin, [19], fits into the category of theory-based models outlined by Bassett et al. [21]. In other words, we test our hypothesis exploring the potentiality of simple neural models, combining these elements without committing to specific biophysical mechanisms. As for the network, we consider a modular version where the oscillations, responsible for the maintenance of the activity, sweep the network probing the modules as a traveling wave.

In the following sections we introduce the theta-alpha interference effect and the modular network model, discuss the results and present some conclusion. The mathematical descriptions of the neural models and the technical details about the computational experiments are left to the end in the Materials and Methods section.

## The model

**Fig 1:**
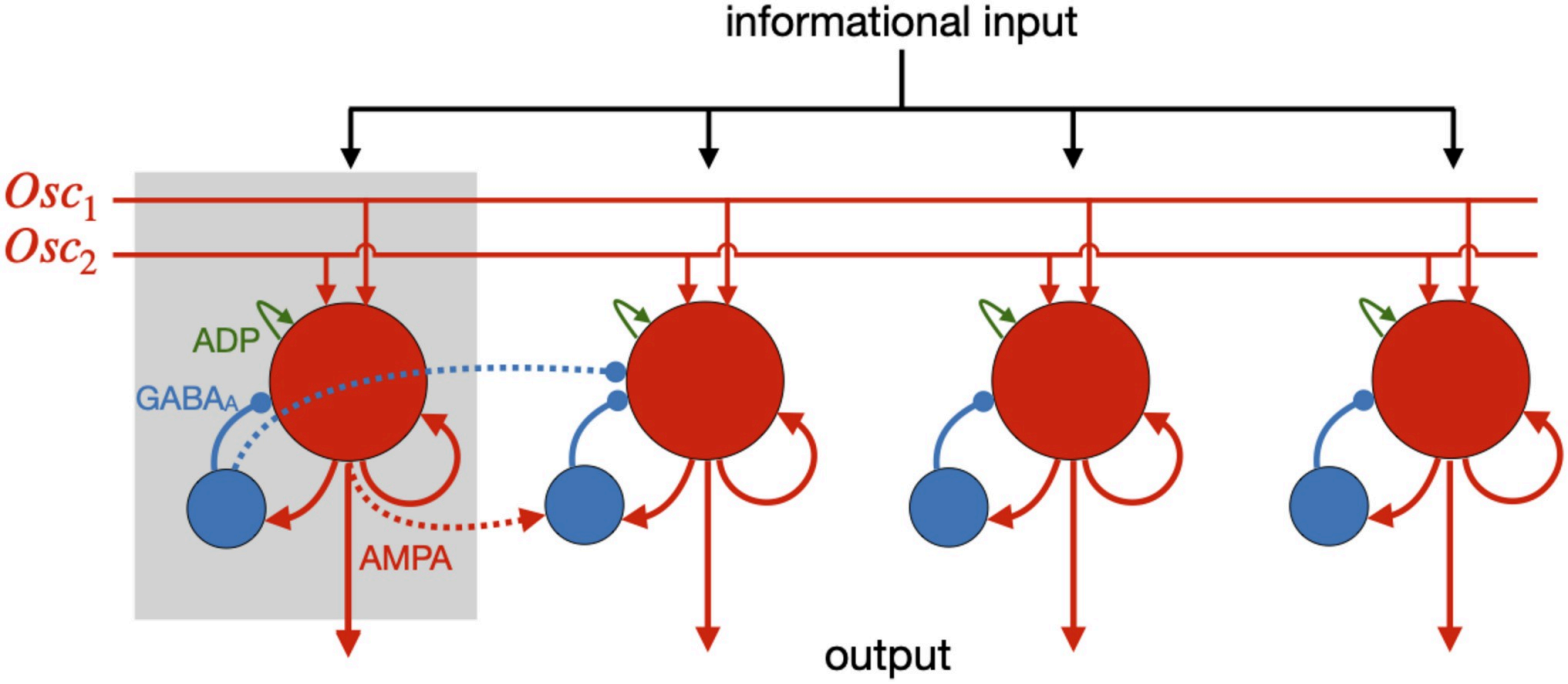
Modular working memory network. The modules are arranged linearly, each one composed by excitatory principal neurons (red circles) and inhibitory interneurons (blue circles). The informational inputs reach all modules synchronously while the unspecific oscillatory inputs (*Osc*_1_ and *Osc*_2_) are traveling waves and therefore, at a given time, the modules have oscillations with different phases. When in storage mode (maintain) a spatial firing pattern is reactivated for each module, producing the cyclic phenomena observed in the rasterplot of Fig 3.

Here we present the modular version of the Lisman-Idiart theta-gamma model. The idea is to use space to improve the robustness of the model to noise as well as to enrich the possibilities of memory storage and manipulation. For simplicity we consider that the modules are distributed in a linear fashion (see Fig 1). In each module a short-term theta-gamma memory model is implemented as a network of excitatory neurons and inhibitory interneurons, that can perform multiple information storage through the cyclic reactivation of spatial firing patterns due to the combination of depolarizing currents after an action potential (ADP) and the excitatory drive by an unspecific oscillatory input on the theta-alpha frequency range (6–12Hz). For most of the analysis, we utilize a current-based integrate-and-fire neural model (IF-Model) with an artificial resettable afterspike depolarization (ADP), as initially proposed by Lisman and Idiart [7]. Additionally, as a proof of concept, we employ a more biophysically realistic model proposed by Rodriguez et al. [22] for L5 PFC pyramidal neurons, where the ADP is explained in terms of intrinsic calcium dynamics (high-threshold calcium currents and calcium-activated nonspecific cation current) in a Hodgkin-Huxley conductance based model (HH-Model). See Materials and methods.

In addition we consider that the oscillatory inputs are traveling waves in such a way that each module receives an oscillation with a slightly different phase. The evidence of travelling waves in the brain dates back almost to the first human EEG recording (see [23]), but its importance has been recently highlighted by experimental work demonstrating that theta propagates in the hippocampus [24–26], and theta and alpha propagate in the neocortex [27]. For a theoretical review see [28].

As in the original Lisman-Idiart Model, each module is capable of coding a number of overlapping firing patterns. But the modular version allows different items to be simultaneously stored in distinct modules that are scanned periodically by the travelling wave. It combines spatial and temporal properties such that the change in the oscillatory frequency input allows a much richer repertoire of operations for manipulating the information held in working memory.

Dipoppa et al [19] proposed four essential operations for a short-term memory model: load, maintain, block and erase. In this paper we show how each one of these operations can be performed in the context of our model, with the exception of the block operation that is not implemented independently but associated with the erase operation.

In other words, we consider that a network is blocked when it cannot hold information. The theta frequency will be responsible for allowing the load and maintain operations to take place and the alpha oscillations will perform the block/erase operations by interfering with the theta oscillations.

## Results

**Fig 2:**
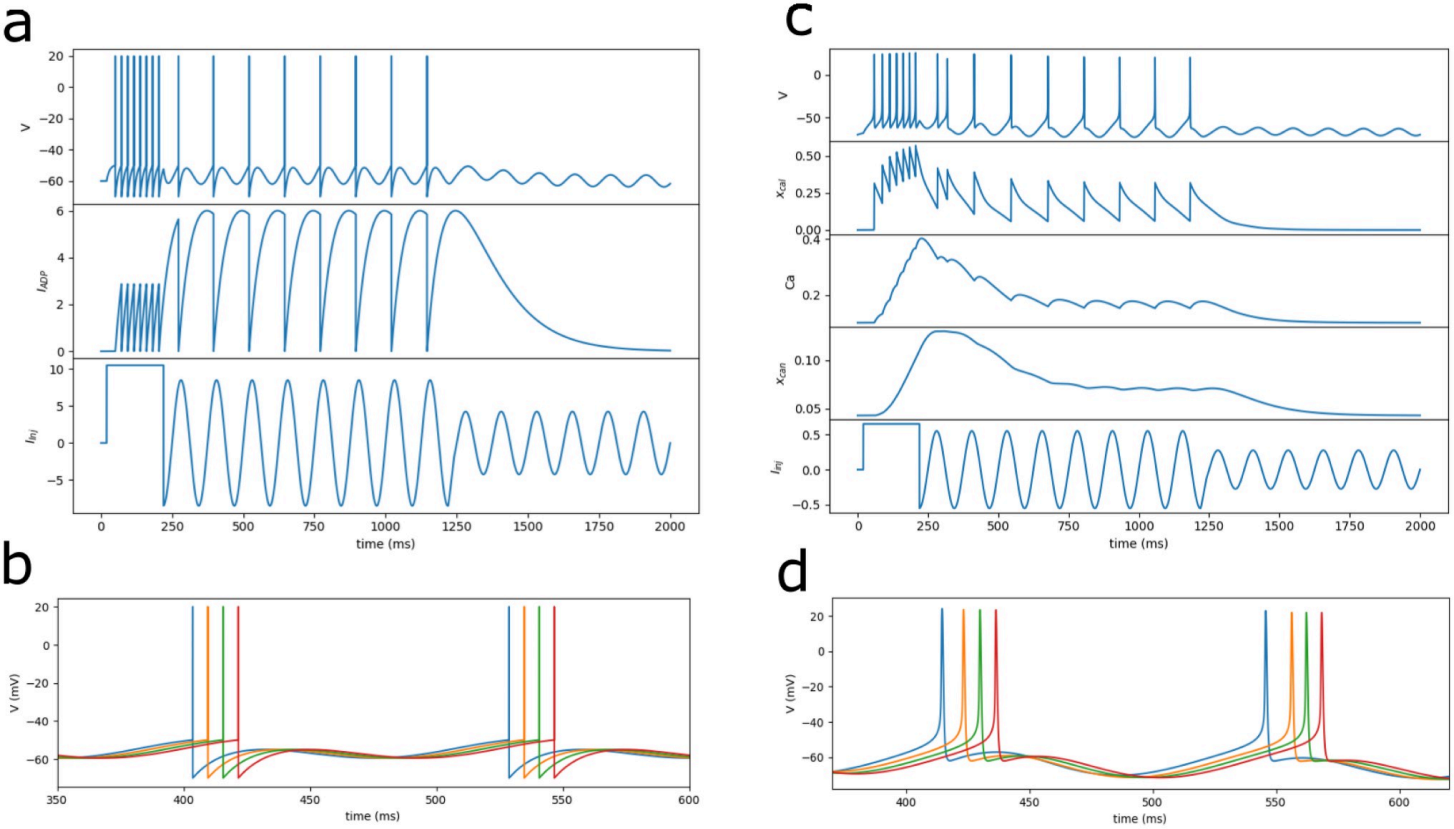
Memory maintenance. The model’s memory behavior can be illustrated as follows: a neuron receives an informational input (square pulse) triggering firing, followed by a subthreshold network oscillation that, on its own, doesn’t induce firing. If the amplitude of the network oscillation is too low the memory is not maintained. In (A), the neuron is an IF-model with stereotyped ADP, and in (C), it is the HH-model [22] with realistic calcium ADP and AHP. Panels (B) and (D) depict the impact of the oscillation phases due to the modularity of the network for both neuron models.

Fig 2 illustrates the basic mechanism governing memory maintenance using both the IF-Model and the more intricate HH-Model. Both neuron models receive a sequence of three inputs: an informational input sufficient to trigger firing, a subthreshold oscillation capable of sustaining firing in neurons that received the informational input but insufficient to cause firing on its own, and an oscillation unable to maintain firing. We observe that the more realistic neuron models, grounded in experimentally observed currents, replicates the behavior exhibited by the simpler model, Fig 2a. We employed the original set of parameters from Rodriguez et al. [22] for calcium currents (CaL, CAN) and calcium dynamics (Ca2), along with the parameters from Golomb and Amitai [29] for spike currents (Na, K), all without requiring further adjustments (refer to Materials and Methods). By supplying sufficient initial external current to activate after-depolarization (ADP), the HH-Model can be driven by an 8*Hz* oscillatory subthreshold current (see Fig 2c), demonstrating bistability through firing once per cycle during the oscillatory phase. The HH-Model proposed by Rodriguez et al. [22] exhibits an additional after-hyperpolarization (AHP) current, absent in our IF-Model. The significance of the AHP current lies in its ability to replicate spike irregularities observed in experimental data [22], this is less relevant in the context of the analysis conducted in this paper where the neurons are reactivated in a much lower frequency. Fig 2b and 2d demonstrate how firing can be temporally segregated in both models if the neurons are driven by oscillations with different phases.

**Fig 3:**
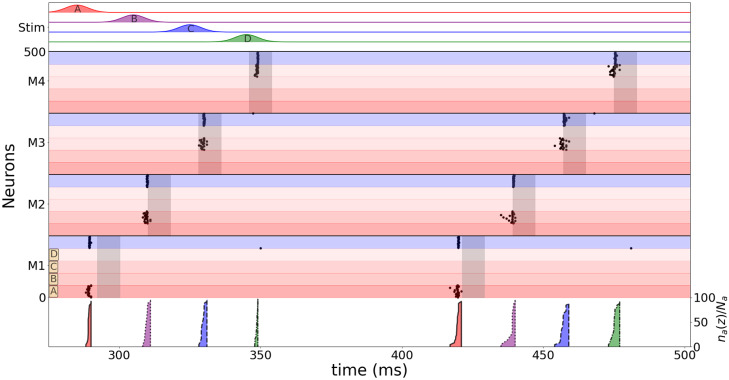
Sequential allocation of items in different modules. On top: four Gaussian stimuli colored and labeled red for A, purple for B, blue for C and green for D. Middle: rasterplot of modules M1, M2, M3 and M4 during two cycles. The excitatory neurons are shown with a red gradient discriminating neurons that codes different stimuli (A-D). Inhibitory neurons are shown in blue. Shaded grey areas indicate the time windows of max neuronal excitability due to the theta wave of (>0.9 max). Bottom: cumulative frequency of the firing of neurons for each module (line style) and each stimulus (color). Label on the right, where a ∈ [A,B,C,D]. Parameters used: *ψ*_*osc*_ = 0.9 rad/module, *f*_*γ*_ = 50 Hz, *f*_*θ*_ = 8 Hz, *ϕ*_*i*_ = 0.8 rad.

Having established, at a qualitative level, the compatibility between the IF-Model and the HH-Model concerning the fundamental mechanism behind our memory model, we now restrict our analysis to the IF-Model. This choice is motivated by its fewer parameters, facilitating a clearer interpretation of the system. Fig 3 illustrates the load operation, that allocates exactly one piece of information per module, when sequential stimuli are fed to the network. On the top we have the input currents of the four stimuli. The currents are Gaussian shaped to account for small asynchronies from the upstream inputs. The activities in each module are represented below with raster plots. The excitatory neurons are shown with a red gradient discriminating neurons that code different stimuli (A-D). Inhibitory neurons are shown in blue. On the bottom, the cumulative frequency for the firing of neurons representing each stimulus for each module are shown. The color code distinguishes each stimulus and the line style distinguishes the modules. Shaded areas represent the peaks of the traveling wave across the network.

**Fig 4:**
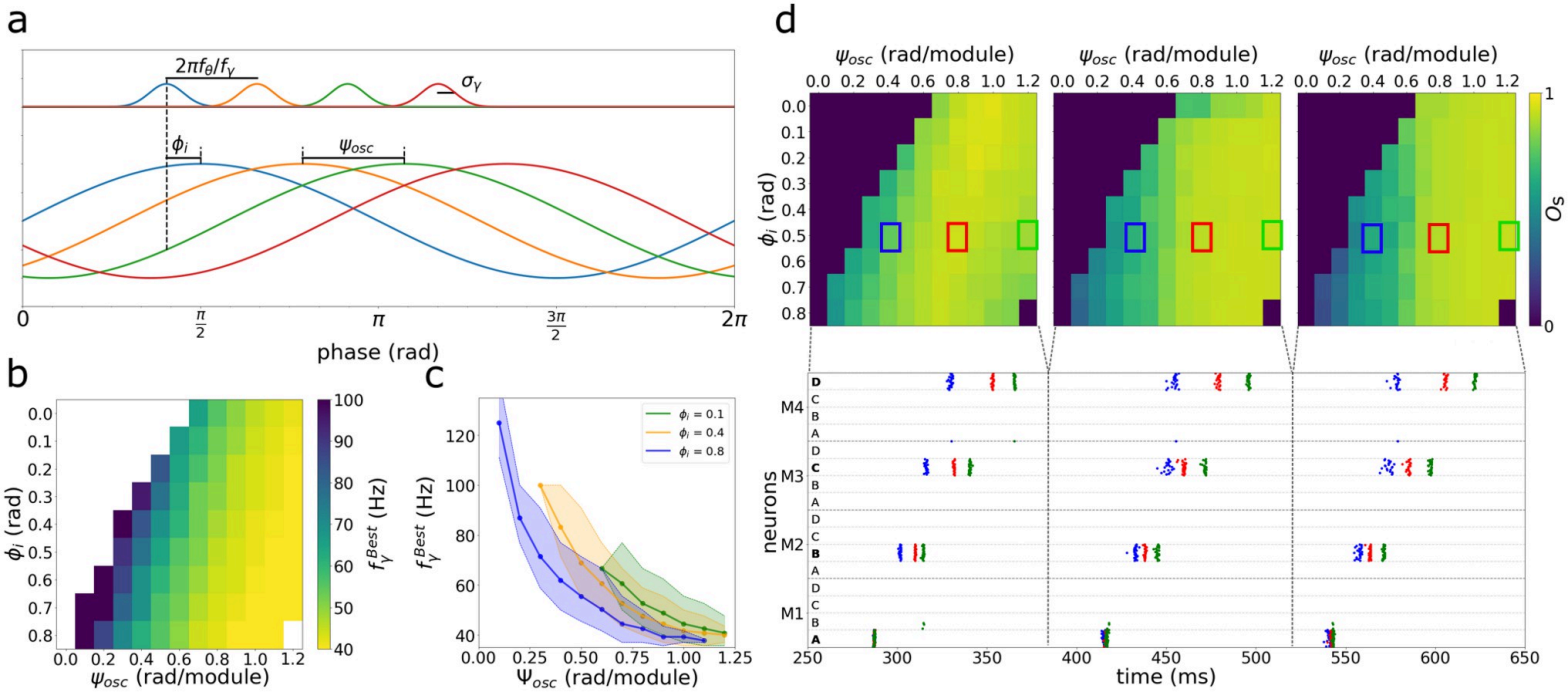
Memory allocation on different modules. A) Definition of *f*_*γ*_, *ψ*_osc_ and *ϕ*_*i*_. Four different stimulus are given equally to all four modules, with a given *f*_*γ*_ frequency, modeled as independent Gaussian currents. *ψ*_osc_ is the phase difference of the external oscillation of two sequential modules. *ϕ*_*i*_ is the phase difference, in module M1, between the oscillations peak and the first input. B) Map of the $f_{\gamma }^{\;Best}$ for a given set of *ϕ*_*i*_ and *ψ*_osc_ when the oscillation is in the theta range *f*_*θ*_ = 8*Hz*. Blank area represents the condition where the four stimulus were not correctly allocated to the four modules. C) $f_{\gamma }^{\;Best}$ dependency on *ψ*_*osc*_ for three values of *ϕ*_*i*_. Shaded areas show the upper and lower values of *f*_*γ*_ that loaded A,B,C and D in M1,M2,M3 and M4. D) *ψ*_osc_ x *ϕ*_*i*_ map of the working memory parameter *O*_*s*_ using the $f_{\gamma }^{\text{Best}}$ during the first three cycles including stimulation (left to right). Three rasterplot samples are shown below in blue, red and green for three different conditions. The map is the mean of 50 repetitions.

In order to better understand the properties of the model after the introduction of the modular structure, we address the question of if there is an optimal delay between stimuli (or an optimal presentation frequency) for the correct allocation of items into different modules. We imagine four items (A through D) being presented to be held in memory at a rate *f*_*γ*_. This is translated in terms of a sequence of Gaussian input currents to the network (Fig 3 top). The Gaussian shapes of the inputs account for a certain degree of asynchrony in the upstream network representing the items and as a whole the input resembles a bout of gamma oscillations of frequency *f*_*γ*_ (Fig 4a). The informational inputs are fed synchronously to all modules of the network, but they reach each of the modules on a different phase of the unspecific oscillatory input due its propagation speed (*v*_osc_). The phase difference between consecutive modules is *ψ*_osc_ = 2*πf*_osc_*d*/*v*_osc_ where *d* is the physical distance between the center of two consecutive modules and *f*_osc_ is the oscillatory frequency, and we make the simplifying assumption that all neurons in a given module are under the same phase. We define the input phase *ϕ*_*p*,*m*_ of the *p*^*th*^ item in the module *m* as the phase difference between the positive peak of the oscillation in that module and the item input time

$$\begin{array}{c} \phi_{p,m}=\phi_{i}-\left(p-1\right)\phi_{\gamma }+\left(m-1\right)\psi_{osc} \end{array}$$

where *ψ*_*γ*_ = 2*πf*_oscd_/*f*_*γ*_ and *ϕ*_*i*_ is the input phase of the first item in the first module (see Fig 4a). The input phase is positive if the stimulus anticipates the peak. Therefore, to evaluate the success of the load operation in different circumstances, three parameters are available: the presentation frequency of the items *f*_*γ*_, the phase difference between modules *ψ*_osc_ and the input phase of the first item in the first module *ϕ*_*i*_.

We consider that the load operation is successful if items presented in a sequence are distributed in an orderly fashion among the different modules preserving sequence information. The easiest of possible orders is to have each item stored in a different module (Fig 3) so that the first item is represented in the first module, the second in the second, and so forth. The success of the load operation, therefore, depends on an optimal temporal match between the timings of the item inputs and the windows of opportunity defined by the positive phases of the oscillatory drive in each of the different modules. This involves adjusting the *f*_*γ*_, *ψ*_osc_ and *ϕ*_*i*_ parameters. We devised a criterion that optimizes *f*_*γ*_ given a choice of *ψ*_osc_ and *ϕ*_*i*_. Fig 4b shows a map where the color indicates the optimal values of $f_{\gamma }^{\text{Best}}$, and where the blank area is the condition where the four stimuli are never allocated correctly (see Materials and methods). Fig 4c has the same information of Fig 4b, but represented as different curves of ($f_{\gamma }^{\text{Best}}\times \psi_{\text{osc}}$) for each *ϕ*_*i*_ condition. In Fig 4d we take the $f_{\gamma }^{\text{Best}}$ for each set of (*ψ*_osc_, *ϕ*_*i*_) and display the multi-item working memory parameter *O*_*s*_ for the three initial cycles including the stimulation (left to right), showing a well defined region for a good working memory performance, exemplified by a rasterplot sample of three different conditions (blue, red and green). *O*_*s*_ is a parameter defined to account for synchronization within modules and asynchronization between modules using only the statistical properties of the neuronal firing in order to evaluate the performance of the multi-item working memory storage (between 0 and 1). S1 Fig shows Fig 4c curve for all *ϕ*_*i*_ conditions and S2 Fig shows Fig 4b and 4c using and alpha oscillation (12 Hz) instead of theta (8Hz).

**Fig 5:**
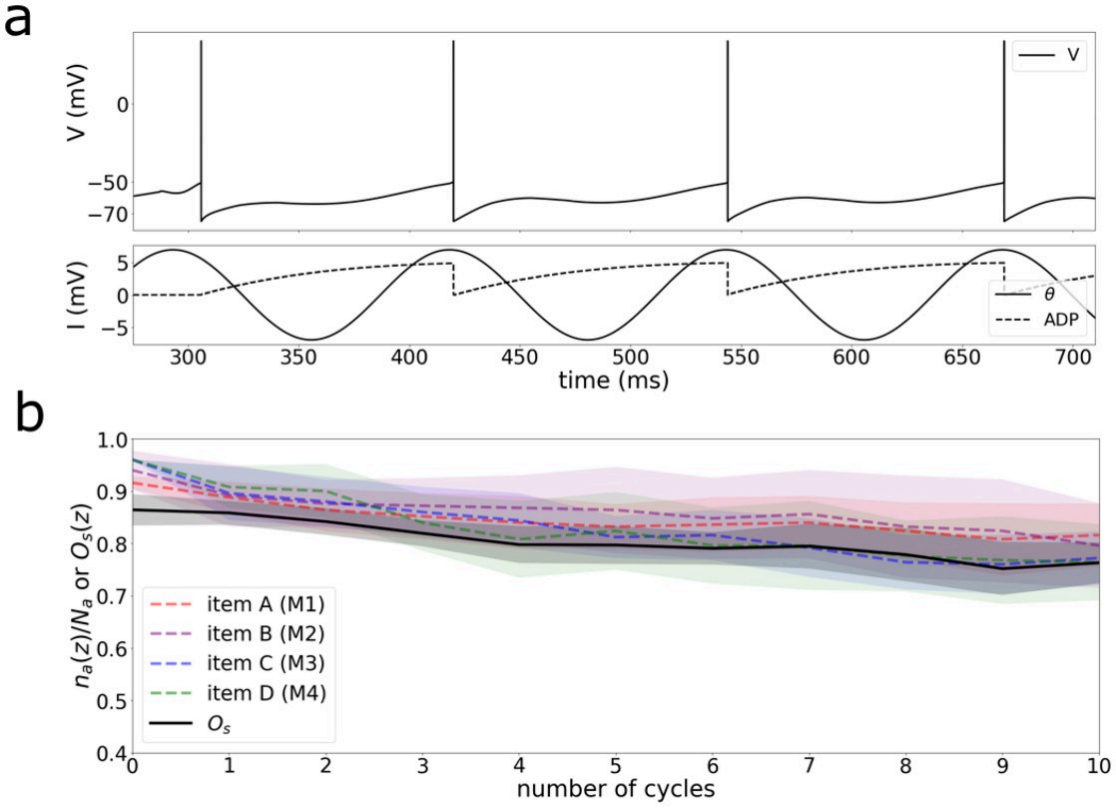
Memory maintenance. A) Basic mechanism for maintenance at the neuronal level: the sum of the oscillatory theta input and the intrinsic afterdepolarization current is sufficient for a cyclic reactivation of the neurons once stimulated. B) Four items, one allocated to each module, are stably maintained in time for several theta cycles. The y-axis accounts for the fraction of active neurons of each item that during each cycle (*n*_*a*_(*z*)/*N*_*a*_, a ∈ [A,B,C,D]) and the *O*_*s*_(*z*) parameter.

The next issue is how well the network maintains information stored through time. In the Lisman-Idiart model for multi-item working memory, the combination of the oscillatory current in theta frequency and the membrane after-depolarization current creates a state of neuronal cyclic reactivation (Fig 5a), therefore neurons activated by a stimulus will be perpetually reactivated.

We consider that the maintenance operation is successfully accomplished if three requirements are met: i) all the neurons representing an item are firing with a good degree of synchrony during memory maintenance ii) each item is stored in a different module so neurons from different items fire asynchronously, iii) memory is reactivated in the first cycle of the unspecific oscillation. Using these criteria, we devised an order parameter (*O*_*s*_(*z*)) that measures the quality of the memory load and maintenance at a given cycle (z) (See Materials and methods). Higher values of *O*_*s*_(*z*) indicate better storage. Fig 5b shows that the four items correctly allocated are stably maintained through time by four different modules.

**Fig 6:**
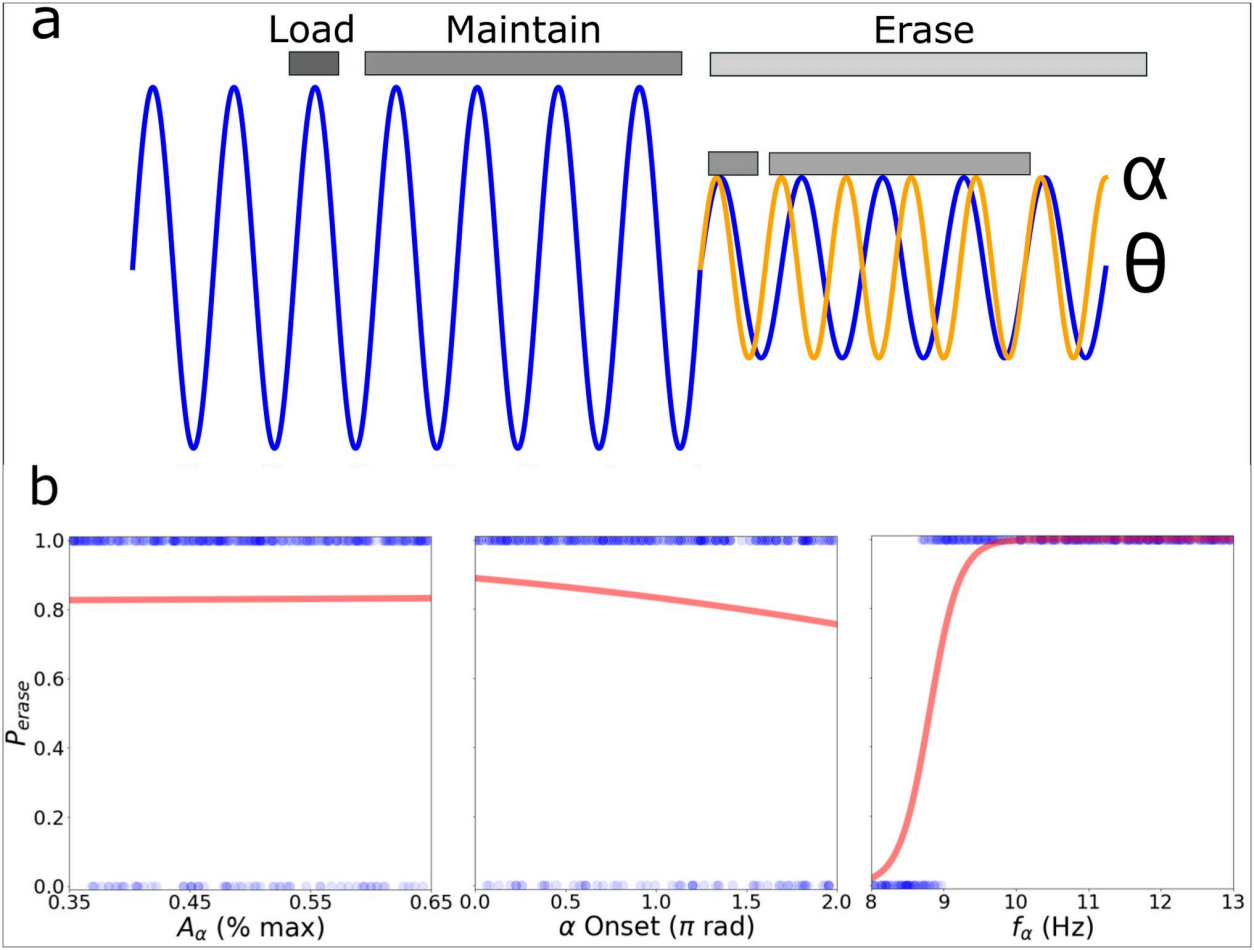
Memory erasing. A) Simulation’s protocol: Load—Information is loaded in the network; Maintain—Information is maintained by reactivation for four oscillatory cycles; Erase—The onset of alpha oscillations occurs and storage measures are made on the next three cycles. B) Multi-item working memory performance measured by the *O*_*s*_ parameter during the first three theta cycles (1’,2’ and 3’) after the onset of alpha. The plots show dependency with the amplitude of alpha, the onset theta phase and the alpha frequency. Thick red lines shows the binned mean, where the markers are the bin’s center and the shadowed area is the standard deviation.

While the power of theta oscillations is correlated with the maintenance of working memory, the power of alpha is thought to actively inhibit irrelevant information on the same tasks. We propose that a possible mechanism for this inhibitory effect is the interference between existing oscillations in the alpha and theta range in the same network. We call it *Oscillatory Interference Hypothesis*. We consider that the combination of alpha and theta frequencies produces a beat, and the cyclic reactivation of the firing patters proposed in Lisman-Idiart ends up impaired and canceled, resulting in an **erase** operation on the working memory buffer (Fig 6a). In order to measure the correlation between the probability of alpha erasing the stored memories and three different parameters of the system (*A*_*α*_, *α* onset and *f*_*α*_), we first calculate the average value of *O*_*s*_(*z*) parameter for the three cycles after alpha onset (*z* = 1′, 2′ and 3′), then binarized using the values of *O*_*s*_ < 0.5 as memory successfully erased (*P*_*erase*_ = 1) and *O*_*s*_ > 0.5 memory not erased (*P*_*erase*_ = 0) and plotted logistic regression fitting curves. Fig 5b shows that the inhibitory role of alpha was independent of the amplitude and the initial onset, but dependent on the specific frequency within the alpha band.

## Discussion

We proposed an adaptation of the Lisman-Idiart model where the neurons are structured in spatial modules and the oscillations are traveling waves. The main reasons for these are (i) to increase the robustness of the model and (ii) to test the gating effect of different external oscillatory inputs. The original model segregated memories through fast-response inhibition, with sequential delays of the firing reactivations [7]. As a result it was unstable to noise and prone to synchronization of neurons belonging to different items. It is important to note that underlying the concept of a multiplexing network, as proposed here, is Singer’s Binding by Synchrony hypothesis [30]. This hypothesis posits that the integral representation of an item is achieved through the synchronization of neural ensembles encoding its characteristics. For instance, the simultaneous representation in short-term memory of a blue circle and a red square is achieved through the specific synchronization of neurons corresponding to the accurate combination of shapes and colors. In simpler terms, blue neurons should synchronize with circle neurons, while red neurons should synchronize with square neurons. Consequently, synchronizing the ensembles of both items is damaging as it eliminates phase information, making it impossible to know the color associated with each shape.

With the introduction of network modularity and the oscillatory inputs as traveling waves the model becomes more robust. Recent evidence suggest that oscillations in the hippocampus and neocortex are indeed traveling waves [25–27]. There is also overwhelming experimental evidence that the brain has a modular architecture, where neurons connect together forming micro local networks. The new version of the model, therefore, introduces not only phase but also space as part of the code. Since lists of items can come in any order we also assume that the modules have enough capacity to represent many items and that a specific item can be represented in different modules. This also solves the problem of storing similar items with overlapping neural representations since they will be active in different modules during multi-item storage.

In the simulated experiments we show that even if four stimuli are given equally to four modules, the excitatory dynamic created by a theta traveling wave allows the correct allocation of one memory item in each module. The optimal frequency to load a sequence of items is related to the speed of the traveling wave, that binds the theta and the gamma oscillations. This frequency is approximately the one in which the gamma period matches the difference in time between the phases of theta oscillations of two sequential modules being smaller as the global inhibition increases. In our model, the stimulus presentation should be interpreted as presented to the neural circuit and not the individual, being preprocessed by other brain regions and temporally compressed before being sent to the working memory system. Recent studies investigating theta and alpha oscillations as traveling waves indicate a phase difference of 2*π* rad/cm in the rat hippocampus [24], 0.1*rad*/*cm* in the human hippocampus [25] and 0.3 − 1.25*rad*/*cm* in the human neocortex [27], suggesting that our model would fit better on a human neocortical surface circuit, with a spatial scale of centimeters in and between modules. Our model indicates that there is a wide range of parameter allowing the theta and gamma oscillations to create the multi-item working memory storage. The blank area of the map of Fig 3b represents a more complex condition where more than one item is coded on the same module or the same item is coded by two different modules. Since every module has all the features of the Lisman-Idiart model, they could operate as local short-term buffers in case there is no interaction between modules. Regarding evidence about the role of the direction of propagation of the travelling waves in working memory [27], one possibility is that the coherence of the propagation of the travelling waves could recruit more or less modules, turning on the spatial features or maintaining just the local ones.

We use the same maintenance mechanism proposed on the Lisman-Idiart model, where stimulated neurons present afterdepolarization currents and the sum with the oscillatory theta input allows a cyclic reactivation. So, the same neurons once stimulated will fire again on each theta cycle. The same patterns activated by the stimuli A,B,C and D are repeated on each cycle for the modules M1, M2, M3 and M4.

The Oscillatory Interference Hypothesis showed to be a plausible mechanism for the blocking role of the alpha oscillations. Considering a condition where the alpha and theta oscillations compete for power (similar amplitudes). Alpha was able to disrupt the working memory performance in the model independently of its amplitude (Fig 6b, left panel). The effect also was relatively insensitive to the phase during the onset of alpha (Fig 6b, middle panel). As for the frequency values there is a sharp transition where, for a theta ≃ 8Hz, values of alpha frequency above 10Hz effectively erased the working memory buffer (Fig 6b, right panel). The measures taken used the combination of three cycles to ensure the time needed for the system to show long lasting and stable behaviour.

**Fig 7:**
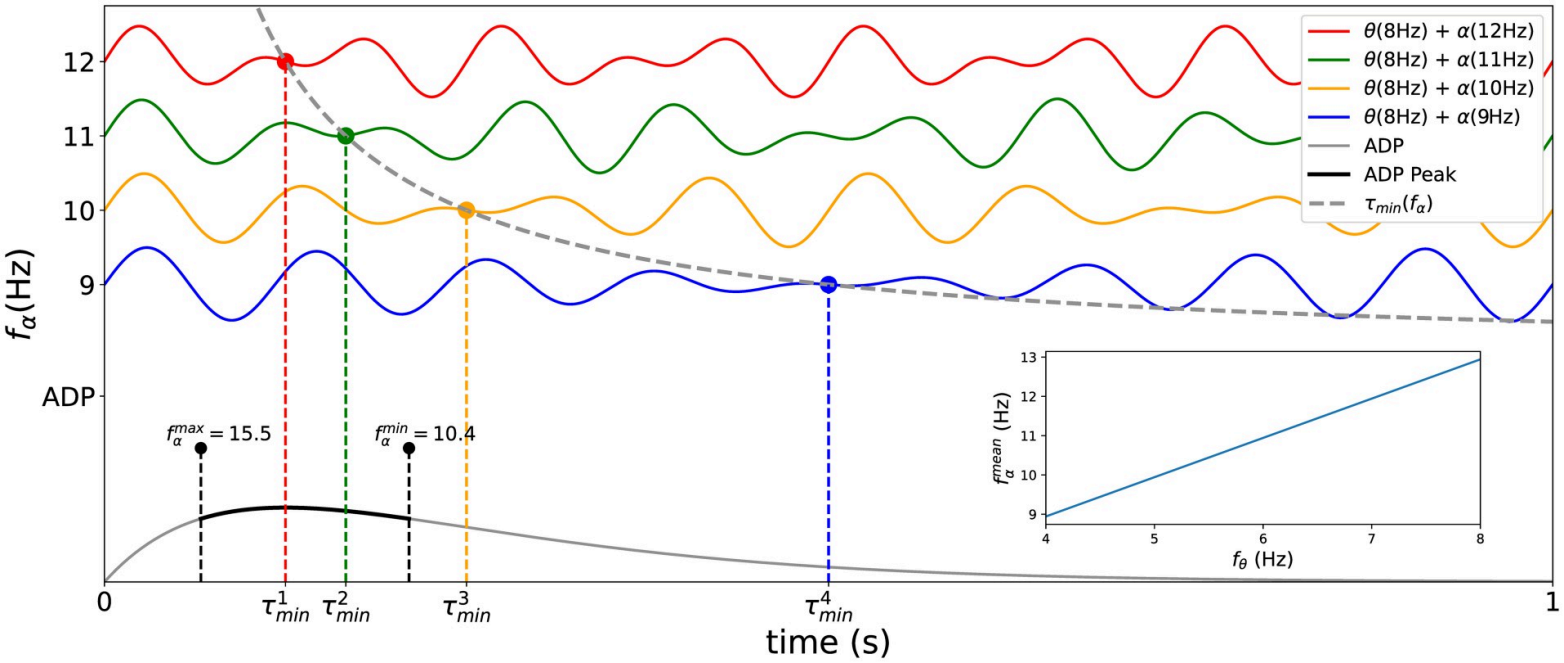
Alpha inhibitory mechanism. A) Beat, in red, produced by an theta (8Hz) and alpha oscillation (10 Hz), dashed black lines, starting synchronized with phase = 0. B) Values of alpha (and higher frequency bands) could produce a beat where the valley coincides with the next two theta peaks, for theta = 8Hz. C) Mean possible alpha (${\bar{f}}_{\alpha }=f_{\alpha }^{max}-f_{\alpha }^{min}$)for other values of *f*_*θ*_.

In our model alpha disrupts working memory by interfering with theta. The result is a beat profile that produces a long lasting period of low amplitude oscillations (Fig 7). Using a condition where both alpha and theta are synchronized at phase = 0 during alpha onset, it is possible to determine which values of alpha (and higher frequency bands) could produce a beat where the valley coincides with the peak of the ADP (*I*_*ADP*_ > 0.85*A*_*ADP*_). Fig 7 top colored curves shows two of the four beatings (red and green) with minimums ($\tau_{min}^{1}$ and $\tau_{min}^{2}$ coinciding with ADP’s peak (ADP shown as the bottom curve, peak in bold) for theta = 8Hz. The analytical curve for the *τ*_*min*_ is computed as half of the inverse of the beat frequency (see Materials and methods) and is showed as dashed gray line. The min and max alpha frequency that coincides with the ADP’s peak is shown closed to the ADP’s curve in black (10.4 Hz and 15.5 Hz, mean value 12.95 Hz). For other values of *f*_*θ*_, the inserted plot shows the dependence between the average value for alpha ($f_{\alpha }^{mean}=\frac{f_{\alpha }^{max}-f_{\alpha }^{min}}{2}$) and theta frequency. This could explain the alpha frequency dependency found during our simulations.

Concerning the potential physiological validity of the derived properties within the IF-Model discussed here, we take initial steps towards a more realistic neural implementation. We showed that, at the fundamental level of memory maintenance, both the IF-Model and the more realistic HH-Model proposed by Rodriguez et al. [22] exhibit similar behavior. The intrinsic currents responsible for the HH-Model’s after-spike depolarization (CaL, CAN) produce an effect akin to the IF-Model’s more stereotyped ADP current. Both exhibit an asymmetric shape, characterized by a faster rise than decay, with amplitudes falling within the range of approximately 2.5 to 15mV. While Rodriguez et al. characterizes the ADP phenomenon as an after-burst depolarization, we consider every depolarization that emerges after each spike (See Fig 2). However, much exploration is needed within the broader parameter space of the HH-Model.

## Conclusion

In this paper, we discussed a possible mechanism behind the effect that oscillations in the alpha range appear to have in cognitive tasks that demand that subjects disregard parts of the external stimuli. We do it in the light of the theta-gamma model proposed by Lisman-Idiart. In this model memory maintenance depends on two factors. The first is an intrinsic excitability caused by recent activity that tags neurons associated with a given memory. The second is an oscillatory input, in the theta range, that is sub-threshold for neurons that are not part of the memory but can drive the tagged neurons back to activity keeping them in oscillatory persistent activity, since there is a resonance effect between the time course of the excitability and the oscillatory input. According to the logic of the model, memory erasure could be accomplished by either eliminating the neural intrinsic response to firing or by disrupting the oscillatory input that refreshes the memory. We propose that an efficient way to disrupt the oscillation is by gating to the circuit an oscillation in the alpha range that will be superimposed over the existing theta oscillation causing amplitude modulations with the exact time scale to prevent the tagged neurons from refreshing their excitability. Although the same effect could be easily accomplished by reducing the power of the theta oscillations, in this paper we subscribe to the view that oscillations are natural attractors for the biological neural networks and preventing them may be more energetically costly than just combining them [31]. In future work we aim to explore the interplay between oscillations and item similarity in long-term memory. This interaction has the potential to mitigate the impact of noise in the model. Noise disrupts firing patterns, causing neurons that should be firing together to desynchronize and synchronizing those that should not. Recurrent connections play a vital role in counteracting these tendencies, thereby preserving the integrity of neural ensembles that collectively represent the same memory. Once the parameter intervals for the basic IF-Model are established, a pivotal next step is to find equivalent parameter intervals in more realistic neural models, such as the one proposed by Rodriguez et al. [22], and subsequently compare them with physiologically realistic parameters.

## Materials and Methods

### Network

We consider a network composed by *N* neurons, of which *N*_*ex*_ are principal excitatory and *N*_*inh*_ are inhibitory interneurons. The network is divided in *M* spatial modules, with *N*_*a*_/*M* neurons of each type, *a* = *ex*, *inh*. The neural connectivity depends on the modules the neurons belong to, as well as the oscillatory inputs, with sequential oscillatory phase-differences producing the effect of a travelling wave. The stimulus input and the output occur simultaneously for all modules.

### Connections

The network has the connectivity matrix presented in the S3 Fig, where the synaptic strengths are randomly generated by an uniform distribution between 0 and *W*_*type*_ that depends on the kind of connection.

$$\begin{array}{c} W_{ab}∼U\left(0,W_{type}\right) \end{array}$$ (1)

where *W*_*ab*_ is the connection from the presynaptic neuron to the postsynaptic neuron and *U*(0, *W*_*type*_) is a uniformly distributed random number between 0 and *W*_*type*_. *W*_*type*_ can be Global (*W*_*EI*_, *W*_*IE*_) or Modular (*W*_*EE*_, *W*_*EI*_, *W*_*IE*_).

### Integrate-and-fire neuron model

The neurons are modeled as current based integrate and fire, given by the equation

$$\begin{array}{c} \tau_{m}\frac{dV}{dt}=-\left(V-V_{r}\right)+\sum_{i}I_{i} \end{array}$$ (2)

where *V* is the membrane potential, *V*_*r*_ is the resting potential, *τ*_*m*_ is the membrane time constant and the last term is the sum over the *i* input currents. The potential is reset to a hyperpolarized value *V*_*reset*_ after passing the firing threshold of *V*_*threshold*_, and stays unable to fire again for refractory time of *t*_*refractory*_.

The total post-synaptic input received by the neuron “i” due to the firing of other neurons “j” in a given time “t” is

$$\begin{array}{c} I_{i}^{ps}\left(t\right)=\sum_{j=1}^{N}W_{ij}\sum_{s=1}^{n_{j}\left(t\right)}P\left(t-t_{j}^{\left(s\right)}\right)\; \end{array}$$ (3)

where *W*_*ij*_ is the synaptic weight, *n*_*j*_(*t*) is the number of spikes fired by the jth neuron up to time *t*, and $t_{j}^{\left(s\right)}$ are the spike times, and the individual input P is

$$\begin{array}{c} P\left(t\right)=H\left(t\right)e^{-t/\tau_{psp}} \end{array}$$ (4)

where *H* represent a Heaviside function. The principal excitatory neurons have an afterdepolarization potential that is reset for each new spike.

$$\begin{array}{c} I_{i}^{ADP}\left(t\right)=A_{ADP}\;(\frac{t-t_{i}^{*}}{\tau_{ADP}})\;e^{-\left(t-t_{i}^{*}\right)/\tau_{ADP}+1}\;H\left(t-t_{i}^{*}\right) \end{array}$$ (5)

where $t_{i}^{*}<t$ is the last spike of cell *i* before time *t*. The informational stimulus are modeled as Gaussian pulses

$$\begin{array}{c} I_{i}^{inf}\left(t\right)=A_{inf}\;\delta_{i\in A}\;e^{-{\left(t-t_{A}\right)}^{2}/\left(2\sigma_{\gamma }^{2}\right)} \end{array}$$ (6)

where *A*_*inf*_ is the input amplitude, *t*_*A*_ the average time the stimulus was presented to the network and *δ*_*i* ∈ *A*_ indicates that only neurons linked to the information pattern A receive the inputs. The oscillatory inputs are given by the sinusoidal function

$$\begin{array}{c} I_{i}^{osc}\left(t\right)=A_{osc}\;\text{sin}\left(2\pi f_{osc}t+\left(m_{i}-1\right)\psi_{osc}\right) \end{array}$$ (7)

where *A*_*osc*_ is the amplitude, *f*_*osc*_ is the frequency, *ψ*_*osc*_ is a phase-shift creating a travelling wave effect and *m*_*i*_ = 1, 2, …, *M* is the module index of the *ith* neuron. The oscillatory power is modeled as all external oscillations come from the same source, meaning that the total power is a constrain condition for the system.

### Noise

We consider only additive noise and introduce it in the simulations as a variability in the firing threshold for each neuron

$$\begin{array}{c} V_{threshold,i}=-50+\eta_{i} \end{array}$$ (8)

where

$$\begin{array}{c} \eta_{i}∼N\left(\mu_{noise},\sigma_{noise}\right) \end{array}$$

is a normally distributed random variable with mean *μ*_*noise*_ and standard deviation *σ*_*noise*_ that is drawn independently for each neuron after each new spike. This is a strategy to decrease simulation time, valid for the IF-Model in the regime of low-frequency spiking (< 10Hz). Within this range, pure white noise added to the threshold is equivalent to more complete treatment of noise as long as its correlation decay time is significantly shorter than the theta oscillation period.

### Neurophysiological realistic neuron model

Neuronal model adapted from L5 PFC pyramidal neurons from Rodriguez et al. [22]. Neurons are Hodgkin-Huxley neurons described by

$$\begin{array}{c} C\frac{dV}{dt}=-\left(I_{L}+I_{Na}+I_{K}+I_{CaL}+I_{CAN}+I_{AHP}\right)+I_{Inj} \end{array}$$ (9)

The Leak current *I*_*L*_ is written as

$$\begin{array}{c} I_{L}=g_{L}\left(V-V_{L}\right) \end{array}$$ (10)

The sodium current *I*_*Na*_ is defined by

$$\begin{array}{c} I_{Na}\left(V,h\right)=g_{Na}m_{\infty }^{3}\left(V\right)h\left(V-V_{Na}\right) \end{array}$$ (11)

with

$$\begin{array}{ccc} \frac{dh}{dt} & = & \left[h_{\infty }\left(V\right)-h\right]/\tau_{h}\left(V\right)\\ m_{\infty }\left(V\right) & = & {\left\{1+exp\left[-\left(V-\theta_{m}\right)/\sigma_{m}\right]\right\}}^{-1}\\ h_{\infty }\left(V\right) & = & {\left\{1+exp\left[-\left(V-\theta_{h}\right)/\sigma_{h}\right]\right\}}^{-1}\\ \tau_{h}\left(V\right) & = & 0.37+2.78*{\left\{1+exp\left[-\left(V-\theta_{ht}\right)/\sigma_{ht}\right]\right\}}^{-1} \end{array}$$ (12)

The potassium current *I*_*K*_ is defined by

$$\begin{array}{c} I_{K}\left(V,n\right)=g_{K}n^{4}\left(V-V_{K}\right) \end{array}$$ (13)

with

$$\begin{array}{ccc} \frac{dn}{dt} & = & \left[n_{\infty }\left(V\right)-n\right]/\tau_{n}\left(V\right)\\ n_{\infty }\left(V\right) & = & {\left\{1+exp\left[-\left(V-\theta_{n}\right)/\sigma_{n}\right]\right\}}^{-1}\\ \tau_{n}\left(V\right) & = & 0.37+1.85*{\left\{1+exp\left[-\left(V-\theta_{nt}\right)/\sigma_{nt}\right]\right\}}^{-1} \end{array}$$ (14)

The high-threshold calcium current *I*_*CaL*_ is determined by the following equations

$$\begin{array}{c} I_{CaL}={\bar{g}}_{CaL}x_{CaL}^{2}\left(V-V_{CaL}\right) \end{array}$$ (15)

with the activation variable *x*_*CaL*_ satisfying

$$\begin{array}{ccc} \frac{dx_{CaL}}{dt} & = & \frac{x_{CaL}^{\infty }\left(V\right)-x_{CaL}}{\tau_{CaL}\left(V\right)}\\ \tau_{CaL}\left(V\right) & = & 10^{\;\alpha_{CaL}+\beta_{CaL}V}\\ x_{CaL}^{\infty }\left(V\right) & = & \{1+\text{exp}{\left[-\left(V-V_{1/2,CaL}/K_{CaL}\right]\right\}}^{-1} \end{array}$$ (16)

The calcium-activated nonspecific cation current *I*_*CAN*_ has the following equation

$$\begin{array}{c} I_{CAN}={\bar{g}}_{CAN}\;x_{CAN}\;\left(V-V_{CAN}\right) \end{array}$$ (17)

with the activation *x*_*CAN*_ depending on the intracellular calcium concentrations as

$$\begin{array}{ccc} \frac{dx_{CAN}}{dt} & = & \frac{x_{CAN}^{\infty }\left(Ca\right)-x_{CAN}}{\tau_{CAN}\left(Ca\right)}\\ \tau_{CAN}\left(Ca\right) & = & \frac{1}{\alpha_{CAN}*Ca+\beta_{CAN}}\\ x_{CAN}^{\infty }\left(Ca\right) & = & \frac{\alpha_{CAN}*Ca}{\alpha_{CAN}*Ca+\beta_{CAN}}. \end{array}$$ (18)

The hyperpolarizing current *I*_*AHP*_ is described by

$$\begin{array}{c} I_{AHP}={\bar{g}}_{AHP}\;x_{AHP}^{2}\;\left(V-V_{AHP}\right) \end{array}$$ (19)

where de activation *x*_*AHP*_ follows

$$\begin{array}{ccc} \frac{dx_{AHP}}{dt} & = & \frac{x_{AHP}^{\infty }\left(Ca\right)-x_{AHP}}{\tau_{AHP}\left(Ca\right)}\\ \tau_{AHP}\left(Ca\right) & = & \frac{1}{\alpha_{AHP}*Ca+\beta_{AHP}}\\ x_{AHP}^{\infty } & = & \frac{\alpha_{AHP}*Ca}{\alpha_{AHP}*Ca+\beta_{AHP}}. \end{array}$$ (20)

The calcium concentration dynamics is described by

$$\begin{array}{c} \frac{dCa}{dt}=-\frac{1}{2F}\frac{Surf}{Vol}I_{CaL}+\frac{Ca_{0}-Ca}{\tau_{Ca}} \end{array}$$ (21)

where

$$\begin{array}{c} \frac{Surf}{Vol}=r_{1}^{-1}{\left(1-\frac{r_{1}}{r_{0}}+\frac{r_{1}^{2}}{3r_{0}^{2}}\right)}^{-1} \end{array}$$ (22)

The injection current is modeled in three modes as

$$I_{inj}=\{\begin{array}{cc} A_{event} & \text{if}\;\;20ms<t<220ms\\ A_{osc}^{delay}\;\text{sin}\left(2\pi f_{osc}^{delay}t+\psi_{osc}^{delay}\right) & \text{if}\;\;220ms<=t<1220ms\\ \left(A_{osc}^{delay}/2\right)\;\text{sin}\left(2\pi f_{osc}^{delay}t+\psi_{osc}^{delay}\right) & \text{if}\;\;1220ms<=t \end{array}$$

The parameters used are summarized in Table 2.

### Metrics for loading performance

In order to evaluate the best frequency of stimuli presentation *f*_*γ*_, for loading information into the network given the parameters (*ψ*_osc_, *ϕ*_*i*_), we simulated the loading cycle (zero cycle) of our network varying *f*_*γ*_ between 100Hz and 33.33Hz. We then counted the number of activated cells for each item for each module *n*_*a*,*m*_ where *a* ∈ [*A*, *B*, *C*, *D*] and *m* ∈ [*M*1, *M*2, *M*3, *M*4]. We consider that, in a first approximation, a frequency is suitable for loading if the order of the stimuli is preserved in the modules. In other words, if the first item is the winner (the most active) in the first module, the second item is the winner in the second module, and so on. Mathematically a binary loading suitability can be written as

$$\begin{array}{c} \ell \left(f|g\right)=\prod_{i}\prod_{j\neq i}H\left[n_{i,i}\left(f\right)-g\;n_{i,j}\left(f\right)\right] \end{array}$$ (23)

where *H*[⋅] is the Heaviside function, the level *g* > 1 is a parameter that controls how bigger the winners must be (for instance, *g* = 2 indicates that the winner has to be at least twice the runner up), and the indexes *i*, *j* are numerical indexes representing items and modules considered here in equal number. We assume that the most suitable frequency (the best *f*_*γ*_) for loading information for a given (*ψ*_osc_, *ϕ*_*i*_) as the average

$$\begin{array}{c} f_{\gamma }^{\text{Best}}\left(\psi_{\theta },\phi_{i}|g\right)=\frac{\sum_{f}\ell \left(f|g\right)f}{\sum_{f}\ell \left(f|g\right)}. \end{array}$$ (24)

When ∑_*f*_* ℓ*(*f*|*g*) = 0 there is no suitable frequency, at level *g*, and the result is represented by a blank in Fig 4b.

### Metrics for performance maintenance

We developed an order parameter that can account for the storage properties of the system. These are: neurons from a given stored item need to be synchronized, while stay asynchronized with neurons from other items. So, for the *z*^*th*^ reactivation cycle and a stored item *A*, the measure for the synchronization within a memory item is

$$\begin{array}{c} O_{A}^{Syn}\left(z\right)=\frac{n_{A}\left(z\right)}{N_{A}}\;{\left[1-{\left(\sqrt{2}\;\frac{\sigma_{A}\left(z\right)}{\Delta t}\right)}^{\beta_{s}}\right]}_{+} \end{array}$$ (25)

where *n*_*A*_(*z*) is the number of active neurons in the ensemble that represents item *A* at the *z*^*th*^ cycle, *N*_*A*_ is the total number of neurons in the ensemble that represents item *A*, *σ*_*A*_(*z*) is the standard-deviation of the firing times of the *n*_*A*_(*z*) neurons, Δ*t* is the time between the reactivation of two sequential items, *β*_*s*_ is a control parameter that punishes the standard-deviation increase. The measure for asynchronization between two memory items *A* and *B*, in the *z*^*th*^ cycle is

$$\begin{array}{c} O_{AB}^{Asyn}\left(z\right)=\phi (\frac{|\left\langle t_{A}\left(z\right)\right\rangle -\left\langle t_{B}\left(z\right)\right\rangle |}{\Delta t}) \end{array}$$ (26)

where

$$\begin{array}{c} \left\langle t_{A}\left(z\right)\right\rangle =\frac{1}{n_{A}\left(z\right)}\sum_{i}t_{A,i}\left(z\right) \end{array}$$

with *t*_*A*,*i*_(*z*) the firing times for the individual neurons representing item *A* and

$$\begin{array}{c} \;\;\;\;\;\phi \left(x\right)=\{\begin{array}{cc} 0 & \text{if}\;\;x=0\\ x^{\beta_{a}} & \text{if}\;\;x\in (0,1)\\ 1 & \text{if}\;\;x\geq 1 \end{array} \end{array}$$

where *β*_*a*_ a parameter controlling *ϕ*’s non linearity.

Our order parameter, therefore, it the multiplicative combination of the average over items of both measures, in a given cycle *z*,

$$\begin{array}{c} O_{s}\left(z\right)=[\frac{1}{M}\sum_{A}O_{A}^{Syn}\left(z\right)][\frac{2}{M(M-1)}\sum_{A,B>A}O_{AB}^{Asyn}\left(z\right)]\;\;. \end{array}$$ (27)

### Metrics for erasing performance

In order to assess alpha’s capacity of disrupting the stored memories, we performed 1200 simulations randomly varying three alpha parameters: *α* amplitude (*A*_*α*_, between 0.35 and 0.65 max), *α* onset (between 0 and 2*π* rad of *θ* ongoing oscillatory phase) and *α* frequency (*f*_*α*_, between 8 and 13Hz). To evaluate alpha’s disrupting performance, we first calculate the average value of *O*_*s*_(*z*) parameter for the three cycles after alpha onset (*z* = 1′, 2′ and 3′), then binarized using the values of *O*_*s*_ < 0.5 as memory successfully erased (*P*_*erase*_ = 1) and *O*_*s*_ > 0.5 memory not erased (*P*_*erase*_ = 0) and plotted logistic regression fitting curves.

### Analytical *τ*_*min*_ curve

We computed the analytical *τ*_*min*_ curve presented on Fig 6 as the half of the inverse of the beat frequency, which represents half of the beating period or the time to achieve the beating minimum.

$$\begin{array}{c} \tau_{min}=\frac{1}{2f_{beat}}=\frac{1}{2}\frac{1}{|f_{\alpha }-f_{\theta }|} \end{array}$$ (28)

### Simulations and data analysis

We used Euler’s method with step size dt = 0.01 ms for solving numerically the neurons differential equations. The simulations were written in C programming language and data analysis and graphic production were made with Python.

### Parameters

Parameters are shown in Tables 1 and 2.

**Table 1:** Overview of parameters. Top: fixed parameters held constant throughout the simulations. Bottom: variable parameters uses more than one value or a range of values.

Fixed Parameters	
Network	*N* = 500
	*N*_*ex*_ = 400
	*N*_*inh*_ = 100
	*M* = 4
Neurons	*τ*_*me*_ = 15 ms
	*τ*_*mi*_ = 2 ms
	*τ*_*ADP*_ = 140 ms
	*A*_*ADP*_ = 7 mV
	*V*_*rest*_ = -60 mV
	*V*_*thresh*_ = -50 mV
	*V*_*reset*_ = -70 mV
	*T*_*ref*_ = 3 ms
Psp current	*τ*_*ps*.*e*_ = 1 ms
	*τ*_*ps*.*i*_ = 10 ms
Global Synaptic Weights	*W*_*EI*_ = 1.12
	*W*_*IE*_ = -0.112
Modular Synaptic Weights	*W*_*EE*_ = 0.70
	*W*_*EI*_ = 4.5
	*W*_*IE*_ =-0.8
*O*_*s*_ Parameter	*β*_*s*_ = 1
	*β*_*a*_ = 1
	Δ*t* = 20 ms
Others	*σ*_*noise*_ = 0.5 mV
	*μ*_*noise*_ = 0 mV
	*σ*_*γ*_ = 4 ms
	*dt* = 0.01 ms
Variable Parameters	
Oscillations	*f*_*θ*_ = 4–8 Hz
	*f*_*α*_ = 8–13 Hz
	*A*_*θ*_ = 0.35–0.65 max
	*A*_*α*_ = 0.35–0.65 max
	*ψ*_*osc*_ = 0—1.2 rad/module
	*ϕ*_*i*_ = 0.0—0.8 rad
	*f*_*γ*_ = 1000—33.33 Hz

**Table 2:** Parameters for the neural HH-Model from [22].

Membrane Equation	*C* = 1*μF*/*cm*^2^
Leak Current(*I*_*L*_)	*g*_*L*_ = 0.05*mS*/*cm*^2^
	*V*_*L*_ = −70*mV*
Sodium Current (*I*_*Na*_)	*g*_*Na*_ = 24*mS*/*cm*^2^
	*V*_*Na*_ = 55*mV*
	*θ*_*m*_ = −30*mV*
	*σ*_*m*_ = 9.5*mV*
	*θ*_*h*_ = −53*mV*
	*σ*_*h*_ = −7*mV*
	*θ*_*ht*_ = −40.5*mV*
	*σ*_*ht*_ = −6*mV*
Potassium Current (*I*_*K*_)	*g*_*K*_ = 3*mS*/*cm*^2^
	*V*_*K*_ = −90*mV*
	*θ*_*n*_ = −30*mV*
	*σ*_*n*_ = 10*mV*
	*θ*_*nt*_ = −27*mV*
	*σ*_*nt*_ = −15*mV*
High-threshold Calcium Current(*I*_*CaL*_)	${\bar{g}}_{CaL}=0.0045mS/cm^{2}$
	*V*_*Cal*_ = 150*mV*
	*V*_1/2,*CaL*_ = −12*mV*
	*K*_*CaL*_ = 7*mV*
	*α*_*CaL*_ = 0.6
	*β*_*CaL*_ = −0.02*mV*^−1^
Calcium-activated Nonspecific Cation Current(*I*_*CAN*_)	${\bar{g}}_{CAN}=0.025mS/cm^{2}$
	*V*_*CAN*_ = 30*mV*
	*α*_*CAN*_ = 0.0056*μM*^−1^*ms*^−1^
	*β*_*CAN*_ = 0.0125*ms*^−1^
Afterhyperpolarizing Current (*I*_*AHP*_)	${\bar{g}}_{AHP}=0.2mS/cm^{2}$
	*V*_*AHP*_ = −90*mV*
	*α*_*AHP*_ = 0.05*μM*^−1^*ms*^−1^
	*β*_*AHP*_ = 0.2*ms*^−1^
Calcium Dynamics (*Ca*)	*F* = 96500*C*/*mol*
	*r*_0_ = 4*μm*
	*r*_1_ = 0.025*μm*
	*Ca*_0_ = 0.1*μM*
	*τ*_*Ca*_ = 100*ms*
Injetion Current (*I*_*Inj*_)	*A*_*event*_ = 0.65
	$A_{osc}^{delay}=0.55$
	$f_{osc}^{delay}=8Hz$
	$\psi_{osc}^{delay}=0rad$
Euler’s Time Step	0.01*ms*

## Supporting information

S1 FigComplementary $f_{\gamma }^{\;Best}$ curves.A) $f_{\gamma }^{\;Best}$ vs *ψ*_*osc*_ for the complete set of *ϕ*_*i*_. B) Mean between *ϕ*_*i*_ conditions.(TIF)

S2 Fig Load operation using alpha. Load operation using alpha 12 Hz instead of theta 8 Hz. Similar plot as Fig 3B and 3C.
(TIF)

S3 Fig Connectivity specification. A) List of excitatory and inhibitory connections. B) Scheme of Global and Modular connections. C) Connectivity matrix for the network. The y-axis represent the presynaptic neurons and the x-axis the postsynaptic neurons. The excitatory and inhibitory neurons are grouped together for convenience.
(TIF)
