# Supplementary figures and images for: Mechanistic role of alpha oscillations in a computational model of working memory
Source: PLoS One. 2024 Feb 8;19(2):e0296217. doi: 10.1371/journal.pone.0296217 (PMC10852337; doi:10.1371/journal.pone.0296217)

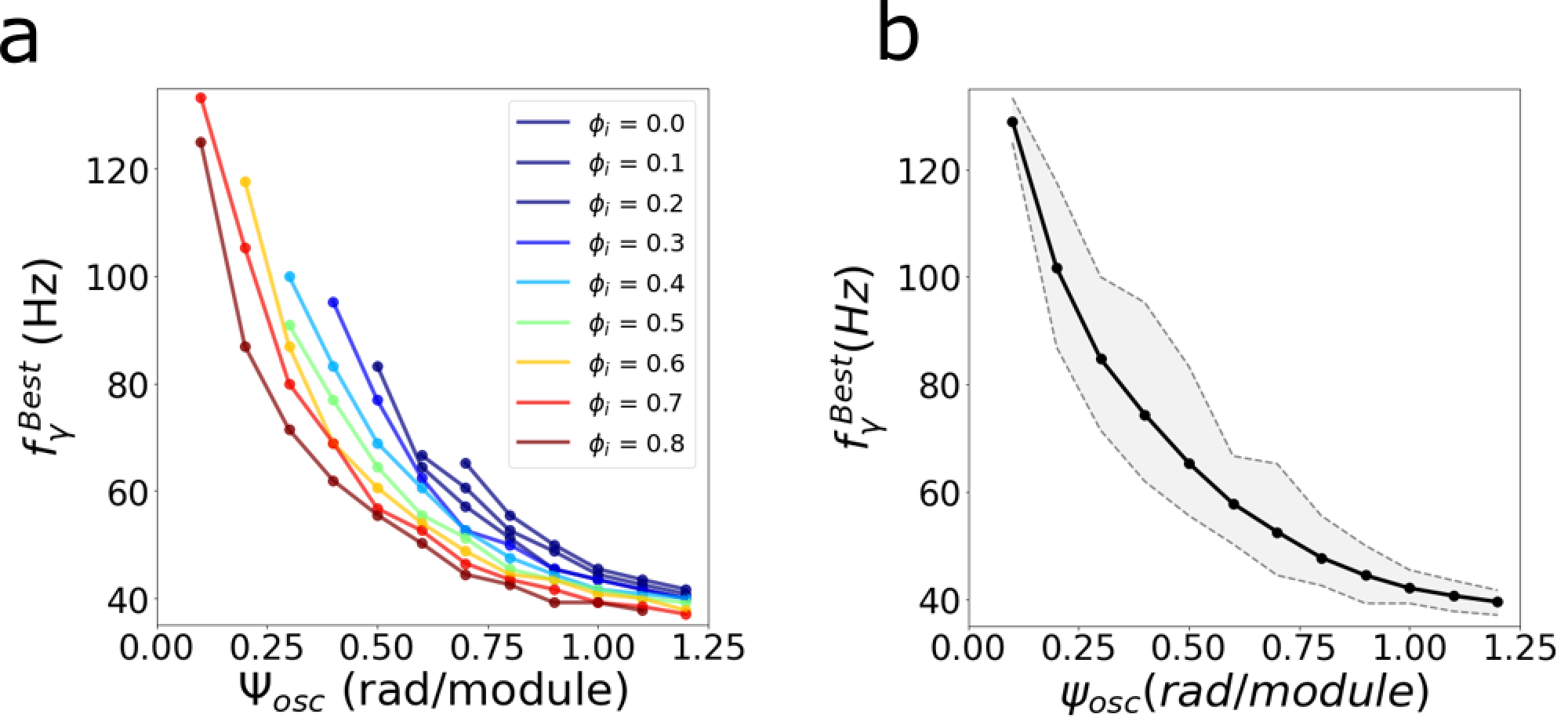

Supplement: S1 Fig — A) fγBest vs ψosc for the complete set of ϕi. B) Mean between ϕi conditions. (TIF) [file pone.0296217.s001.tif]

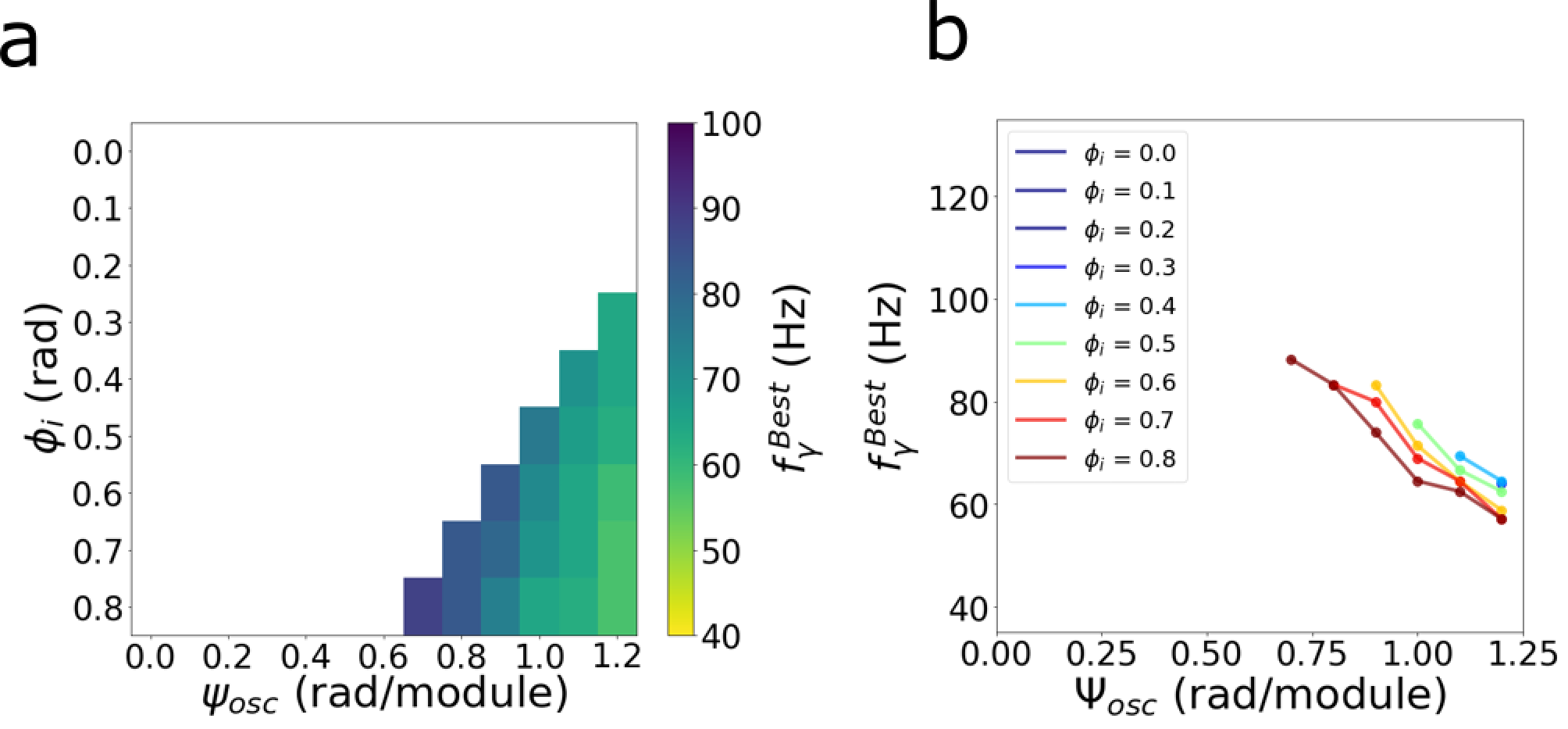

Supplement: S2 Fig — Load operation using alpha 12 Hz instead of theta 8 Hz. Similar plot as Fig 3B and 3C. (TIF) [file pone.0296217.s002.tif]

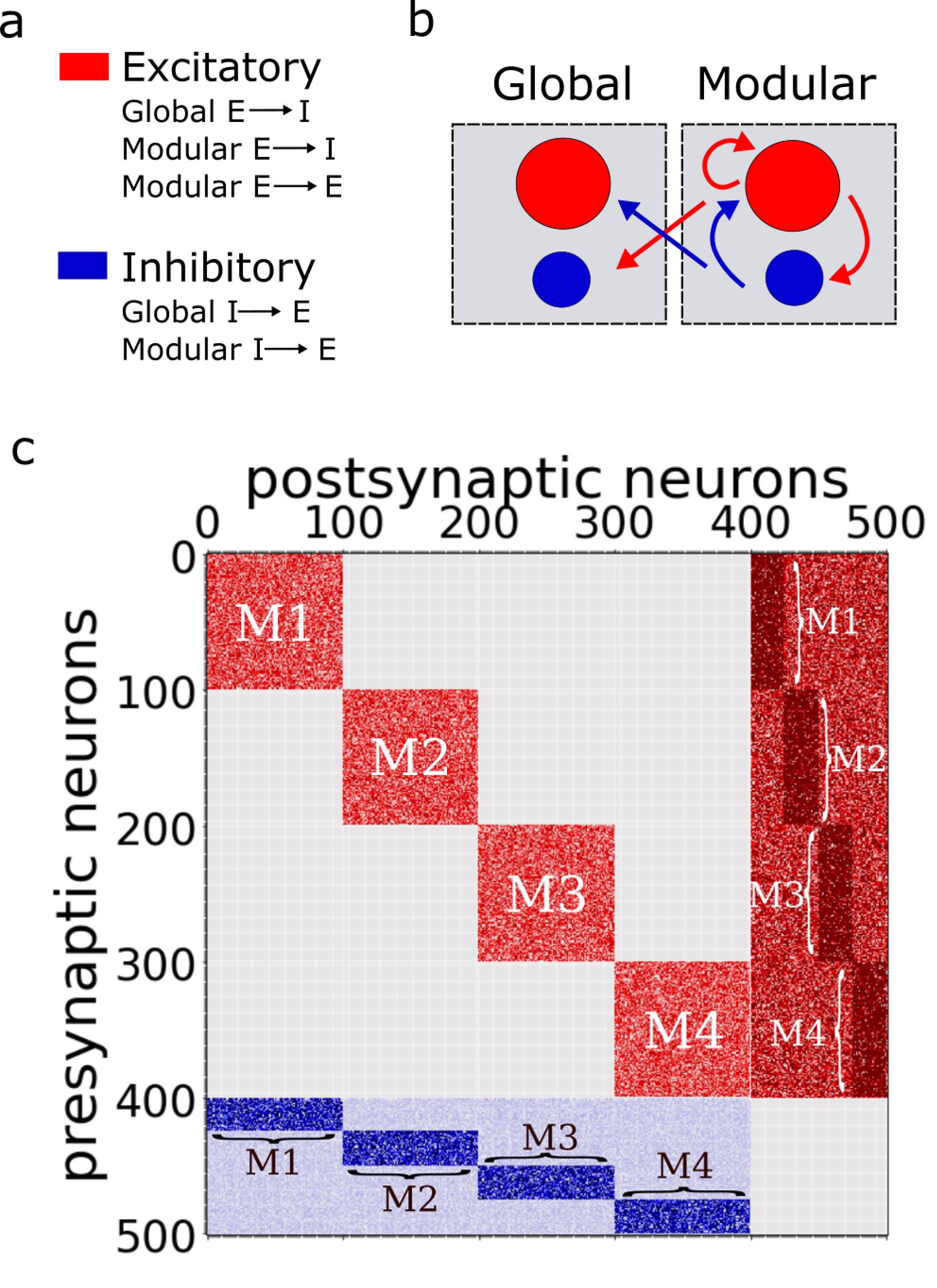

Supplement: S3 Fig — A) List of excitatory and inhibitory connections. B) Scheme of Global and Modular connections. C) Connectivity matrix for the network. The y-axis represent the presynaptic neurons and the x-axis the postsynaptic neurons. The excitatory and inhibitory neurons are grouped together for convenience. (TIF) [file pone.0296217.s003.tif]
